# Mathematical Evaluation of the Amino Acid and Polyphenol Content and Antioxidant Activities of Fruits from Different Apricot Cultivars

**DOI:** 10.3390/molecules16097428

**Published:** 2011-09-01

**Authors:** Jiri Sochor, Helena Skutkova, Petr Babula, Ondrej Zitka, Natalia Cernei, Otakar Rop, Boris Krska, Vojtech Adam, Ivo Provazník, Rene Kizek

**Affiliations:** 1 Department of Chemistry and Biochemistry, Faculty of Agronomy, Mendel University in Brno, Zemedelska 1, CZ-613 00 Brno, Czech Republic; 2 Department of Biomedical Engineering, Faculty of Electrical Engineering and Communication, Brno University of Technology, Kolejni 4, CZ-612 00 Brno, Czech Republic; 3 Department of Food Technology and Microbiology, Faculty of Technology, Tomas Bata University in Zlin, Namesti T. G. Masaryka 275, CZ-762 72 Zlin, Czech Republic; 4 Department of Fruit Growing, Faculty of Horticulture, Mendel University in Brno, Valticka 337, CZ-691 44 Lednice, Czech Republic; 5 Central European Institute of Technology, Brno University of Technology, Technicka 3058/10, CZ-616 00 Brno, Czech Republic

**Keywords:** apricot, amino acids, polyphenolics, antioxidant activity, statistical analysis

## Abstract

Functional foods are of interest because of their significant effects on human health, which can be connected with the presence of some biologically important compounds. In this study, we carried out complex analysis of 239 apricot cultivars (*Prunus armeniaca* L.) cultivated in Lednice (climatic area T4), South Moravia, Czech Republic. Almost all previously published studies have focused only on analysis of certain parameters. However, we focused on detection both primary and secondary metabolites in a selection of apricot cultivars with respect to their biological activity. The contents of thirteen biogenic alpha-L-amino acids (arginine, asparagine, isoleucine, lysine, serine, threonine, valine, leucine, phenylalanine, tryptophan, tyrosine, proline and alanine) were determined using ion exchange chromatography with UV-Vis spectrometry detection. Profile of polyphenols, measured as content of ten polyphenols with significant antioxidant properties (gallic acid, procatechinic acid, *p*-aminobenzoic acid, chlorogenic acid, caffeic acid, vanillin, *p*-coumaric acid, rutin, ferrulic acid and quercetrin), was determined by high performance liquid chromatography with spectrometric/electrochemical detection. Moreover, content of total phenolics was determined spectrophotometrically using the Folin-Ciocalteu method. Antioxidant activity was determined using five independent spectrophotometric methods: DPPH assay, DMPD method, ABTS method, FRAP and Free Radicals methods. Considering the complexity of the obtained data, they were processed and correlated using bioinformatics techniques (cluster analysis, principal component analysis). The studied apricot cultivars were clustered according to their common biochemical properties, which has not been done before. The observed similarities and differences were discussed.

## 1. Introduction

Plenty of studies focused on the determination of bioactive compounds and their biological values in fruits have been published in the last decade [1,2,3,4,5]. Fruits are fundamental constituents of nutrition, widely discussed due to their high contents of elements, amino acids and primary and secondary metabolites. Among the mentioned biochemicals, secondary metabolites are directly responsible for many of the beneficial effects of fruits on human health. The chemoprotective activity of these compounds, especially polyphenols, refers to the demonstrated ability to protect other biomolecules from chemical damage leading to loss of function [6,7,8,9,10,11]. In addition, these compounds can significantly affect the effect of therapy and the progress of diseases by interfering with the pharmacokinetics of pharmaceuticals [12]. The connections between consumption of fruit (and vegetables) and reduced risk of cancer is still being investigated [13,14]. Due to this fact, fruit is also widely discussed as a functional food [15]. Fruits are usually cultivated in a wide range of cultivars, which differ not only in growth abilities, adaptability to given climatic conditions and yield, but also in chemical composition. In addition, new fruit cultivars are still being cross-bred and selected, however the possible beneficial effects on human health are unknown due to the lack of investigations on the contents of biologically active compounds, including primary and secondary metabolites.

Nutritional value is among the most used parameters for fruit characterization. It is usually defined as the content of basic compounds, such as sugars, lipids and amino acids [16]. Amino acids occur in fruits in free form, or more commonly bound in peptides, proteins and non-protein compounds. Only nine amino acids (tryptophan, valine, leucine, isoleucine, lysine, threonine, methionine, phenylalanine, and histidine) are considered to be essential for the correct functioning of many crucial biochemical pathways [17]. They support the immune system, digestion, the function of the nervous system [18,19] and many others [20]. Qualitative and quantitative occurrence of amino acids in fruits together with vitamins and trace elements determine their organoleptic properties, nutrition and biological values [21,22,23,24].

Apricots (*Prunus armeniaca* L.) are trees which are widely cultivated around the World. Apricot fruits contain many bioactive compounds which are beneficial to human health. Therefore, it is not surprising that wild apricots are used in some folk medicines, especially in Asia [25]. These beneficial components of apricots include amino acids, minerals, carotenoids, phytosterols and polyphenolics, especially flavonoids and anthocyanins, which are closely connected with antioxidant activity and biological effects [26,27,28,29,30,31,32,33,34,35,36,37,38]. Simple hydrocarbons and their derivatives belonging to the group of terpenes are responsible for the aroma of fruits, however, their biological activity is still largely unknown [39]. Polyphenolics, together with alkaloids, belong to the most discussed class of secondary metabolites. They are found in almost all plant species, but their qualitative and quantitative occurrence is species-specific and unique. They serve as pigments, attractants for pollinators, and they carry out important protective functions due to their antioxidant activity [40,41]. Their oxidation-reduction properties make these compounds possible participants in electron transport pathways. Based on this fact, one may suggest that they can have regulation functions in plant cells [42,43]. They are also connected with the transport of the auxin plant hormones, which participate in fruit ripening processes [44,45]. There are many papers that demonstrate the beneficial effects of these compounds on human health [46,47,48,49]. Their pharmacological characteristics, including antioxidant properties [50,51], which are important for prevention of diseases connected with oxidative stress, such as cardiovascular diseases [52,53], cancer [54,55], neurodegenerative diseases, diabetes and hypertension [53,55,56] have been described.

Determination of antioxidant activity is one of the methods of expression of the presence of compounds with antioxidant properties in samples of interest [51]. Methods of determination of antioxidant activity are most often based on some direct reaction of the studied compound with radicals (extinction or scavenging) [57] or on their reaction with metals [58]. Determination of antioxidant activity may thus be used to express the biological value of fruits. This value depends significantly on the type of polyphenols in the fruits, especially due to the fact that some polyphenols have higher antioxidant activity in comparison with the others, based on the specific structural features, especially the presence and mutual position of hydroxyl groups [59,60,61,62].

In this study, we focused on performing a detailed biochemical profiling of 239 apricot cultivars from the Gene Pool of the Czech Republic (5-8-8, 5-17-103, 6-10-45A, Abu Talibu, Ackermann, Agat, Achrori, A-II 25/65, Alfred, Amos, Ananasnyj Čjuripinskij, Ansu, Antonio Erani, Apricos von nansy, Arzam. Aromatnyj, Aurora, Aviator, Bai-Gon, Beliana, Bergeron A114, Blenheim, Blenheim Orange, C4R8T22, California, Cegledi Bilbor, Celgledi Biror, Cocov, Colomer Nativ, Colov 19, Curtis, Čačansko zlato, Čína, Čudovnys, Dacia, DA-YU-BADA, Docteyr Maccle, Doc. Blatný, Early Blush, Early Gold, Early Ryl, Efekt-22/7, Exherova, Farmingdale, Favorit, Festivalna, Forum, Fruhe von Kitsee, Gergana, Goldcot, Goldrich, Goldtropfen, Goliaš, Gulia, Gvardějskyj, H-14/25, Hacihaliloglo, Harcot, Harglow, Hargrand, Harval, Helena de Rousilion, Hendersen, H-I-9149, H-II 16/1, H-II 19/40, H-II 45/26, Chersonskij, Churmai, Chvang sin, Chvan-Shi-Cong, I 51/00, In-Bez-Sin, Iskra, Ivone Liverani, Jubilejnyj, Julskij, Jvan-Sin, Keczli Mete Rosen, Klosterneuburger, Konzervnyj pozdnyj, Kospotěnskij, Kostinskij, Kostuřenskyj, Krasnošč Nikitskij, Krupnoploda, Krupnoplodá II, Krymsk Medunec, Labert in 4NO, Lajcot, Lakanyj, LE-1075, LE-11/91, LE-1402, LE-2267, LE-3204, LE-3709, LE-4725, LE-5306, LE-5500, LE-5832, LE-5854, LE-6016, LE-6283, LE-7150, LE-7463, LE-858, LE-8711, LE-946, LE-982, LE-985, LE-994, LE-995, Leala, Lebeza, Ledana, Lefreda LE-831, Lefrosta, Legolda, Lejara LE -386, Lemeda, Lenova, Lepana, Leronda, Lerosa, Leskora, Ligeti Orias, Litoral, Ljotčik, Luizet, M 47, M 56, maďarská235C, Magiar Kajszii, Machová, Mai-Huang, Mamaia, Manicot, Marena, Markulesti, Marlen, Martina Bassi, Melitopolskij, Merculešti 17/2, Merkurij, MK 132 Visus Foee, Moi-Schva-Sin, Mold Olimpik, Moldavský krup, Mongold, Murfatlar, Murgab, Nachodská Krajová, Náchodský zázrak, Nansy Aprikose, Nikitskij, NJA-1, NJA-2, NJA-35, NJA-55, Nugget, Orange red, Oranževo Krasnyj, Pacov, Pastyrik, Pentagon, Perfection, PLM.78FIŠR, Poirat, Polgocvetna, Poljus Južnyj, Polonais, Posjolok, Poyer, Pozdní chrámová, Pretendent, Priusaděbnyj, Reliable, Reumberto, Riland, Rival, Rodina, Rotmaler, Rouge de Fourner, Rouge de Rive Saltes, Rouge de Sarnhac. Roxana, Ružová Ranná, Sabinovská LE-220, Saldcot, Scana, Scout, Sem Badem Eric, SEO, SEO-104, Seo-105, SEO-111, SEO-116, SEO-31, SEO-36, SEO-40, SEO-41, SEO-74, SEO-94, Sosed, Stelar, Strepet, Sundrop, Sungiant, Sunglo, Sungold, Šalah, Štepnjak Oranževyj, Tabu, TDB, Tilton, Užgorod, Vardaguin, Vagdaas, Vebama, Veecot, Velikyj, Velita, Velvaglo, Veselka 74/14, Vesna, Vesprima, Vinoslivij, Vivagold, Vnuk Krasnoščokogo, Volšebnyj, Voskij, VP-LE-118, VP-LE-12/6, VP-LE1212, VS22/23, VS-23/164, Vyndrop, Wenatchee, Zard, Zorkij). Contents of amino acids (alanine, aspartic acid, glycine, glutamic acid, histidine, isoleucine, leucine, phenylalanine, proline, serine, threonine, tyrosine and valine), polyphenols (gallic acid, procatechinic acid, *p*-aminobenzoic acid, chlorogenic acid, caffeic acid, vanillin, *p*-coumaric acid, rutin, ferrulic acid and quercetrin) and antioxidant activity were determined for all studied cultivars. The results obtained were processed by statistical and bioinformatics tools with the aim of determining the connections between individual apricot cultivars, their bioactive compounds and analytical methods used for their analysis as the unique way to characterize the gene pool.

## 2. Results and Discussion

### 2.1. Statistical Evaluation

Due to the expected large set of data, we aimed primarily at defining suitable bioinformatics tools for processing them. Multidimensional data analysis was used to treat the very extensive set of data obtained. Combinations of two methods such as principal component analysis (PCA) and cluster analysis (CA) appeared to be the most useful in statistical analysis, where the use of only one method could be insufficient. In addition, the CA method was very useful as a control method for the PCA analysis, in which some information is lost.

#### 2.1.1. Principal Component Analysis (PCA)

Reduction of the number of linear cross-correlations (correlated parameters) to the representative objects, which describe the most important information necessary for data description, was the main objective of the use of this method. The method is based on linear transformation of original characteristics (“symbols”) to new uncorrelated values, which are called principal components. These principal components are characterized by variability, which represents dispersion. By using PCA the method principal components were ranked in accordance with their significance, which means in the accordance with decreasing dispersion [63]. The original characteristics were unable to differentiate between objects (in our case apricot cultivars) due to their minimal or eventually zero dispersion. The degree of dispersion of the values of the original characteristics was determined in the first step of analysis.

Characteristics with lower dispersion less contributed to the final analysis. Therefore, normalization of parameters was made to maintain the major part of information contained in the original data (characteristics). However, in doing so the rate of parasitic, “ghost” information was increased. Due to this fact, it was impossible to reduce the dimensionality of the data. Therefore, normalization based on arithmetic mean or medial of parameters was performed. This disunity had no effect on our resulting analysis because of the mathematical/significance incoherence of features of the characteristics in individual measurements (amino acids, phenolic compounds, antioxidant activity). Selection of k-matrix of the principal components based on the variability of original characteristics followed normalization. The parameter *P* expressing degree of variability of original characteristics was determined in consequence with the following formula [66]:
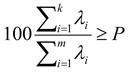

where *P* represents the percentage of variability *k* of the principal components representing m-matrixes of original characteristics, where λ_i_ are values—numbers—symbolizing diagonal particulars of matrix of dispersion of the original characteristics from the mean value. *P* was selected between the ranges from 70% to 90%. The main components were arranged in accordance with percentage of variability. Interpretation of results was carried out in accordance with the results of selected basic components analysis. They were reconstructed using a disperse diagram of component scores. The great advantage of this projection consists in the fact that distances as well as angles between individual objects are preserved. A parameter “component score” was established for the description of rate between individual principal components dispersion and dispersion of original characteristics. Component score represents the scale of principal axes under two-dimensional/tree-dimensional description of results. Two-dimensional presentation enabled evaluation of isolated objects, which means apricot cultivars, which differ extremely in analyses from other cultivars based on representative principal components. Three-dimensional presentation was suitable for evaluation of similarity of individual objects such as apricot cultivars. Three separated clusters of apricot cultivars were closely connected. This fact predicates significant similarity between apricot cultivars. Analyses were supplemented by separation of object into clusters, cluster analysis and colour presentation in three-dimensional graphs of individual clusters.

#### 2.1.2. Cluster Analysis (CA)

Cluster analysis may be profitably used in the cases where objects tend to be separated into clusters. This fact was verified by the PCA method. The CA method works on the principle of projection of analysed objects onto multidimensional space (the number of dimensions depends on the number of measured parameters) with subsequent hierarchical interlinking of object into tree-like structures (dendrograms) based on their mutual distance. This method was beneficial for evaluation of the results of the obtained analyses, because they were arranged in accordance with their similarities into classes (clusters) [64]. Standardized Euclidean distance, which expresses degree of similarity of objects, was used for calculation of mutual distance between individual objects. Euclidean distance *d_E_* is calculated as a hypotenuse of length of rectangular triangle [68]:

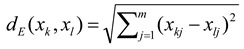

where *k* and *l* are two objects, whose Euclidean distance is determined and represent vertices of triangle at the hypotenuse. The third vertex *j* determines the length of the sides *x_kj_* and *x_lj_* of a rectangular triangle, which is defined for all objects 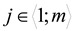. Standardization of distance was performed at the level of characteristics, which means before projection of objects onto space. This was performed with respect to the characteristics with higher degree of variability, because they have higher effect on the degree of similarity.

Figures were standardized in accordance with standard deviation, especially with regard to quality of presentation. Matrixes of distances for all pairs of objects were constructed from determined values of degree of similarity of analysed apricot cultivars. Dendrograms were designed from this matrix using the Unweighted Pair Group Method with Arithmetic Mean (UPGMA method). The closest pair of analysed apricot cultivars was identified. Further, this pair was put together into a node and the length of branch was determined as their average distance. This pair was replaced in matrix by their node connection, which means that one row and one column were eliminated and replaced by the average distance from all remaining analysed apricot cultivars to the node (pair of closely connected apricot cultivars). All objects (apricot cultivars) were hierarchically connected into clusters. Size of matrix was reduced for one row and column in each step of analysis. Clusters were represented in colour in accordance with suitable specific threshold value. Separation of objects (apricot cultivars) using both CA and PCA was performed. Results of both used methods are agreeable. Using of these methods made analysis and classification of obtained data more reliable.

### 2.2. Amino Acids Determination

Amino acids are essential for all life-sustaining processes as the building blocks of peptides and proteins [65,66]. Amino acids are also important for the taste of fruits. They increase the taste of other compounds and they have their own tastes ranging from tasteless (arginine, asparagine, isoleucine, lysine, serine, threonine, valine) to bitter (leucine, phenylalanine, tryptophan, tyrosine) and sweet (proline, alanine). Due to the intensive investigation of amino acids in plant tissues and their translocation within plant bodies, robust analytical methods are needed [67]. Reverse phase chromatography with pre-column derivatization is the most widely used technique for determination of free amino acids in biological materials, including plant tissues [68]. Nonpolar stationary phases mimic the composition of biological membranes [69,70]. Due to this fact, derivatized amino acids may be selectively separated and subsequently analysed. In this study, we used ion exchange chromatography, which is especially used for detection of total amino acids after hydrolysis, coupled with post column derivatization using ninhydrin for determination of alanine, aspartic acid, glycine, glutamic acid, histidine, isoleucine, leucine, phenylalanine, proline, serine, threonine, tyrosine and valine content. The average contents of amino acids determined in the various apricot cultivars are summarized in Table 1. Contents of alanine ranged from to 1,012 to 12,360 mM, aspartic acid 100 to 997 mM, glycine 1,004 to 17,120 mM, glutamic acid 104 to 941 mM, histidine 101 to 833 mM, isoleucine 101 to 982 mM, leucine 53 to 895 mM, phenylalanine 892 to 5,753 mM, proline 104 to 999 mM, serine 110 to 996 mM, threonine 1,107 to 9,984 mM, tyrosine 71 to 607 mM and valine 97 to 1,395 mM. The obtained values were statistically analysed and correlated. The correlation results lacked statistical significance (the highest correlation—0.69—was between proline and lysine). With respect to the results of correlation, these values were not used in the next evaluation.

**Figure 1:**
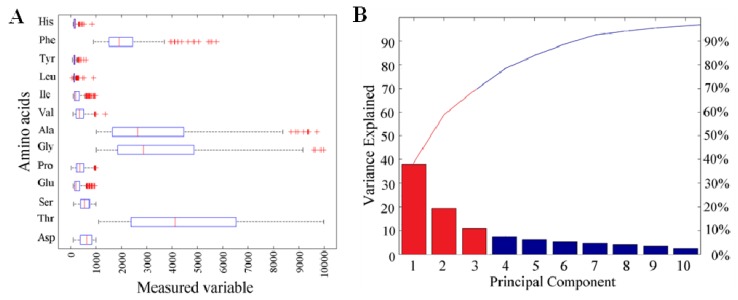
Content of alanine (Ala), aspartic acid (Asp), glycine (Gly), glutamic acid (Glu), histidine (His), isoleucine (Ile), leucine (Leu), phenylalanine (Phe), proline (Pro), serine (Ser), threonine (Thr), tyrosine (Tyr) and valine (Val). Liquid chromatography apparatus AAA 400 (Ingos, Czech Republic) was used for determination of amino acids. (**A**) Box plot graphs of range values of content of amino acids of interest (mM, the average, mean and variance are expressed); (**B**) representation of the content of amino acids by the use of ten principal components.

**Figure 2:**
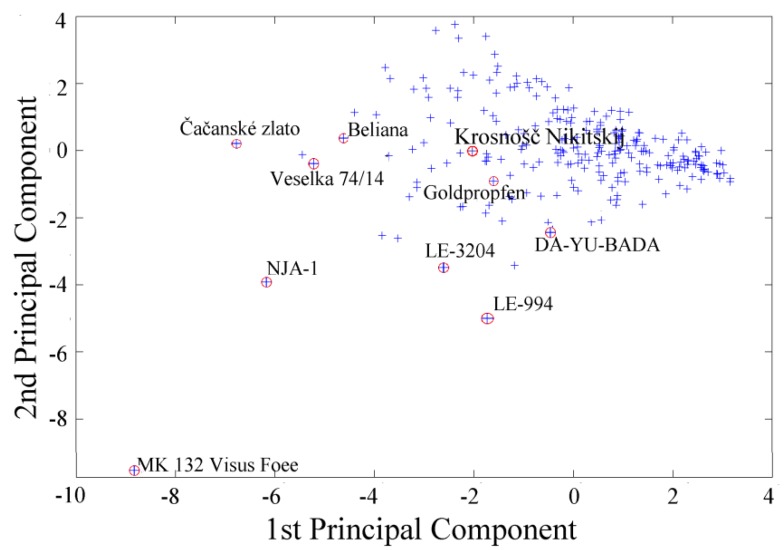
Statistical expression of extreme values of amino acids content represented by two principal components. The marked apricot cultivars had the extreme values markedly different from the others.

One of the most important aims of this work consisted in sorting apricot cultivars in accordance with their amino acids contents due to their importance for nutritional aspects like potential digestibility. The obtained results showed significant differences in contents of individual amino acids in apricot fruits (Table 1). Because of this fact, it is not possible to compare individual cultivars based on total amino acids (as a sum of amino acids), and contents of each amino acid must be compared individually among apricot cultivars. A matrix, where each amino acid had its significance, was constructed. Thanks to this matrix, we were able to determine the contents of amino acids and especially the highest content of amino acids among apricot cultivars. The highest amino acid content was determined in three cultivars: Efekt-22/7, NJA-1 and Čačansko zlato. On the other hand, the lowest content was determined in fruits of cultivars LE-985, Murgab and Vinoslivij. In addition, the statistical evaluation method of multiparametric data was used for the further analysis of the data. For the well-arranged presentation of obtained data, graphic presentation based on combination of principal component analysis (PCA) and cluster analysis (CA) was chosen. Because results of both analyses are not complete proof, both methods are suitable in mutual combination with subsequent control of results (Figure 1 and Figure 2).

Usage of PCA demands that each parameter influence the resulting principal components to an equal extent (representation of dispersions of individual parameters by the form of box plots graphs). Due to representation of results in a box graph (Figure 1A), we can express a range of contents of individual amino acids in apricot cultivars. Box graphs enable well-arranged presentation of obtained results. Figure 1A also shows significant differences in the range values of individual parameters. Due to this fact, it was necessary to normalize parameters with the help of their average values to have the same significance before application of PCA. Normalized values of parameters were recalculated to the values of principal components, thus, decreasing data dimensionality. The figure of representation of the principal components represents 95% of values of measurement by means of ten principal components (Figure 1B). As it is well evident in the figure, three principal components represent more than 70% of primary characters of parameters of measurement and they are suitable for three-dimensional projection of results of measurements (Figure 1B).

**Figure 3:**
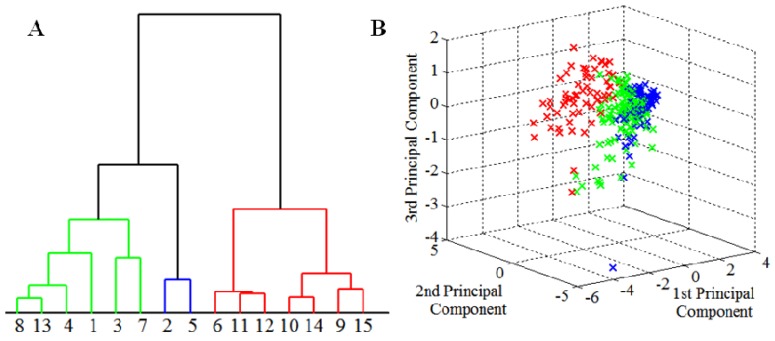
(**A**) Statistical clustering of apricot cultivars according to their content of amino acids. All apricot cultivars were clustered into three clusters; (**B**) Three-dimensional projection of the clusters to distinguish their relations.

Despite the fact that two principal components represent only 60% of input parameters, they are sufficient for denotation of the orientation of extreme values, meaning apricot cultivars which mostly differ from the others. The majority of these extremes fall out of the main cluster of marked objects, as it is clearly evident in Figure 2. Results of 2D projection of extreme values were also confirmed in three-dimensional presentation of three principal components. The whole set of apricot cultivars (gene pool) was divided into three representative clusters. The biggest cluster (red) comprises 80% of all apricot cultivars, which have approximately the same amino acid composition. The rest of the apricot cultivars, almost all extremes from the two-dimensional presentation, are classified into the other clusters. This evaluation was verified using cluster analysis and represented by a simplified dendrogram plot. Due to this way of data presentation, we can clearly identify apricot cultivars with distant values. Using cluster analysis, we were able to suggest a dendrogram which divides individual apricot cultivars into several representative clusters based on mutual similarity of their measured parameters. Clusters classify individual apricot cultivars into groups based on similar characteristics. Figures 3A and B, which utilize concurrent colour marking, demonstrate the results of both methods.

Results of both methods correspond and evaluate extreme and standardized values of measured objects. All apricots were divided into 15 groups as follows:

**Group 1:** 5-17-103, Agat, A-II 25/65, Arzam. Aromatnyj, Blenheim, Cegledi Bilbor, Celgledi Biror, Doc. Blatný, Festivalna, Gergana, Goldrich, Hargrand, Harval, Helena de Rousilion, H-II 45/26, Chersonskij, In-Bez-Sin, Kospotěnskij, Kostuřenskyj, Lakanyj, LE-1402, LE-858, LE-8711, Legolda, Litoral, Markulesti, Moldavský krup, Nugget, Perfection, Polgocvetna, Rodina, Roxana, SEO-105, VP-LE1212, VS22/23, Zard;

**Group 2:** Abu Talibu, Ansu, Apricos von nansy, Aviator, Cocov, Docteyr Maccle, Early Blush, Favorit, Fruhe von Kitsee, Goldcot, Goliaš, H-14/25, Hendersen, Churmai, I 51/00, Ivone Liverani, LE-5306, LE-985, Lebeza, Lejara LE -386, Leronda, Ljotčik, Mamaia, Martina Bassi, Merculešti 17/2, MK 132 Visus Foee, Murgab, PLM.78FIŠR, Poljus Južnyj, Poyer, Sabinovská LE-220, Scout, Sem Badem Eric, SEO-104, SEO-111, SEO-40, SEO-41, Stelar, Sunglo, TDB, Vebama, Vinoslivij, Vnuk, Krasnoščokogo, Volšebnyj, Voskij;

**Group 3:** Amos, Čína, Goldtropfen, Harglow, Lajcot, LE-3204, LE-4725, LE-6283, Ledana, Lenova, Ligeti Orias, Nachodská Krajová, Orange red, Riland, SEO-36, Štepnjak Oranževyj, VS-23/164;

**Group 4:** 6-10-45A, C4R8T22, Colomer Nativ, Early Ryl, Gulia, Krupnoplodá II, Machová, Mold Olimpik, Nikitskij, Polonais, Pretendent, Reliable, Reumberto, SEO-74, Velikyj, Velvaglo, Vesna;

**Group 5:** Ackermann, Bai-Gon, Blenheim Orange, Colov 19, DA-YU-BADA, Hacihaliloglo, H-I-9149, Iskra, Jubilejnyj, Klosterneuburger, Konzervnyj pozdnyj, Kostinskij, Krasnošč Nikitskij, LE-1075, LE-5500, LE-7463, LE-995, Magiar Kajszii, Marlen, Merkurij, Murfatlar, Náchodský zázrak, NJA-35, Pacov, Pozdní chrámová, Rotmaler, Šalah;

**Group 6:** 5-8-8, Alfred, Aurora, Bergeron A114, Dacia, Krymsk Medunec, LE-5854, Lefreda LE-831, Priusaděbnyj, Sundrop, Tabu, Vyndrop;

**Group 7:** Achrori, California, Čudovnys, LE-5832, LE-7150, Leala, Lefrosta, Lemeda, Lepana, Leskora, Manicot, Marena, Poirat, Rouge de Fourner, Veecot, Vesprima, Vivagold;

**Group 8:** Curtis, Exherova, LE-11/91, Mai-Huang, Nansy Aprikose, Pastyrik, Rouge de Rive Saltes, SEO-31, Sosed;

**Group 9:** Harcot, Julskij, Krupnoploda, LE-6016, LE-946, LE-982, Scana, Vardaguin Vagdaas;

**Group 10:** Ananasnyj Čjuripinskij, Efekt-22/7, Farmingdale, H-II 19/40, LE-2267, Posjolok, Rival, Zorkij;

**Group 11:** M 56, Melitopolskij, Moi-Schva-Sin, NJA-55, SEO, Tilton, VP-LE-118, VP-LE-12/6;

**Group 12:** Early Gold, H-II 16/1, Keczli Mete Rosen, Labert in 4NO, LE-3709, Lerosa, maďarská235C, Rouge de Sarnhac, Ružová Ranná, SEO-116, Velita;

**Group 13:** Gvardějskyj, Chvan-Shi-Cong, Jvan-Sin, LE-994, Luizet, M 47, Oranževo Krasnyj, Pentagon, Saldcot, SEO-94, Sungiant, Užgorod, Wenatchee;

**Group 14:** Antonio Erani, Beliana, Forum, Mongold, NJA-1, NJA-2, Sungold;

**Group 15:** Čačansko zlato, Chvang sin, Strepet, Veselka 74/14.

This presentation of the data analysis enables us to determine similar apricot cultivars from the point of view of amino acid content. This has importance for monitoring their biological and nutritional value.

### 2.3. Determination of Selected Polyphenols

**Figure 4:**
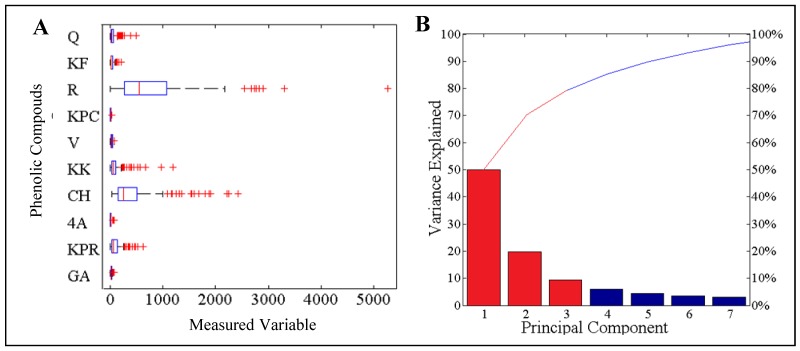
Content of GA-gallic acid; KPR-protocatechuic acid; 4A-*p*-aminobenzoic acid; CH-chlorogenic acid; KK-caffeic acid; V-vanillin; KPC-p-coumaric acid; R-rutin; KF-ferulic acid; Q-quercetin. For the determination of HPLC profile of individual apricot cultivars, high performance liquid chromatography with electrochemical and UV-VIS detection was used. (**A**) Box plot graphs of range values of content of polyphenol of interest (µM, the average, mean and variance are expressed); (**B**) Representation of the content of polyphenols by the use of ten principal components.

In addition, the profile of polyphenols was determined in the fruits of apricot of all 239 apricot cultivars. The following important polyphenols were quantified: gallic acid, procatechuic acid, *p*-aminobenzoic acid, chlorogenic acid, caffeic acid, vanillin, *p*-coumaric acid, rutin, ferrulic acid and quercetrin. These polyphenols are especially important because of their antioxidant properties [71]. Data obtained by the analysis of ten phenolic compounds describing 239 apricot cultivars were evaluated using dispersion analysis and relative representation of values. Output was projected in the form of box plot graphs (Figure 4A). Statistical methods of analysis of multidimensional data are to a large extent equivalent to data obtained in previous analysis of amino acids. As it is well evident, the range of values of individual parameters differs significantly for each one, especially the parameters R (rutin) and CH (chlorogenic acid), which can significantly influence further mathematical data processing. Therefore, parameters were normalised using medians to have the same effect.

Normalised values of parameters were recalculated to the values of principal components in the view of reduction of data dimension (Figure 4B). Figure 4B showing participation of principal components represents 95% of values of measurement through seven principal components. As it is well evident from the figure, three principal components represent almost 80% of parameters of measurement. Therefore, we reduced the data dimensions from ten to three on the level of significance of 80%, which is suitable for further data processing. In comparison with amino acids, phenolic compounds present a higher rate of correlation. Data analysis determined the correlation between ten analysed phenolic compounds as object, but there were no statistically significant correlations between the contents of individual compounds. The most significant correlation was proven between quercetin and rutin (0.73) and between ferrulic acid and *p*-coumaric acid (0.71). Values of correlations between other analysed phenolic compounds were lower than 0.7.

**Figure 5:**
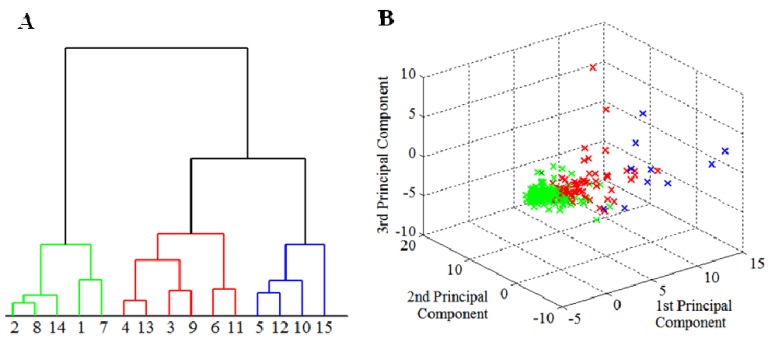
(**A**) Statistical clustering of apricot cultivars according to their polyphenol content. All apricot cultivars were clustered into three clusters; (**B**) Three-dimensional projection of the clusters to distinguish their relations.

Cluster representation by dendrogram and three-dimensional projection of principal components verifies a high rate of linear dependence between the input parameters of measured objects–polyphenols and apricot cultivars (Figures 5A and 5B). Smaller side clusters can be again identified as marginal extremes due to comparison of polyphenol content with the results in the principal group of apricot cultivars. This principal group is formed by 168 apricot cultivars samples, which represent 72% of the all cultivars. They are represented by a green cluster. The smaller red cluster represents approximately 20% deviation from the principal group. The blue cluster indicates the rest of the cultivars.

All apricots were divided into 15 groups as follows:

**Group 1:** 5-8-8, Achrori, A-II 25/65, Ansu, Apricos von Nancy, Arzam. Aromatnyj, Aviator, Beliana, Bergeron A 114, Cegledi Biror, Cocov, Colomer Nativ, Curtis, Čačansko zlato, Daciam, Doc. Blatný, Efekt-22/7 245, Exherova, Farmingdale, Harglow, Helena de Rousilion, H-I.9/49, H-II16/1, H-II19/40, Iskra, Julskij, Klosterneuburger, Kospotěnskij, Kostinskij, Kostuženskij, LE-5306, LE-946, LE-982, LE-994, Leala, Lefrosta, Luizet, Magiar Kajszii, Mold Olimpik, Náchodská Krajová, Oranževo Krasnyj, Pentagon, Poljus Južnyj, Polonais, Poyer, Priusaděbnyj, Reliable, Rotmaler, Rouge de Sarnhac, Růžová ranná, Sabinovská LE-220, Scaha, SEO-105, SEO-40, SEO-41, SEO-74, SEO-94, Strepet, Sundrop, Tabu, Velita, Veselka 74/14, Wenatchee;

**Group 2:** Alfred, Amos, Aurora, H-14/25, Churmai, In-Bej-Sin, Krupnoplodá II, LE-3709, LE-5854, Lerosa, Orange Red, Perfection, PLM.78FIŠR, Rival, SEO-111, Velikyj, Vesna;

**Group 3:** Čína, Chvang sin, SEO-31;

**Group 4:** Ackermann, DA-YOU-BADA, Favorit, Festivalna, Fruhe von Kitsee, Gvarděskyj, Ivone Liverani, Jubilejnyj, Krymsk. Medunec, LE-2267, LE-5500, Ledana, Lefreda LE-831, Lenova, Ligeti Orias, M 47, M 56, maďarská235C, Markulesti, Merculešti 17/2, Moi-Schva-Sin, NJA-2, Nugget, Polgocvetna, Rodina, Rouge de Fournes, Scout, Sosed, Sungold, Štepnjak Oranževyj;

**Group 5:** Early ryl, Harcot, Lemeda, SEO-36;

**Group 6:** Ananasnyj Cjuripinskij, Chvan-Shi-Gong, Labert in 4NO, Lajcot, LE-3204, Reumberto, Šalah, Volšebnyj, Vyndrop;

**Group 7:** 6-10-45A, Agat, Bai-Gon, Blenheim, Blenheim Orange, California, Goldcot, Goldrich, Gulia, Hargrand, Harval, Henderson, I 51/00, Krasnošč Nikitskij, Lakanyj, LE-1075, LE-11/91, LE-5832, LE-6283, LE-7463, LE-985, LE-995, Lebeza, Lepana, Machová, Mai-Huang, Manicot, Marlen, Martina Bassi, Nikitskij, NJA-1, Pastyrik, Pretendent, Riland, Rouge de Rive Saltes, SEO, SEO-116, Stelar, Sungiant, Sunglo, TDB, Užgorod, Vardaguin Vagdaas, Veecot, Vegama, Velvaglo, Vinoslivij, Vivagold, VP-LE-118, VS22/23, VS-23/164;

**Group 8:** Docteyr Maccle, Hacihaliloglo, LE-1402, LE-4725, LE-6016, Leronda, Marena, Mongold, Murfatlar, Murgab, Nansy Aprikose, NJA-35, Roxana, SEO-104, Vesprima, Vnuk Krasnoščokogo, VP-LE1212, Zard, Zorkij;

**Group 9:** Cegledi Bilbor, Colov 19, Goldtropfen, Keczli Mete Rosen, LE-7150, LE-858, Legolda, Moldavský krup, NJA-55, Pozdní chrámová;

**Group 10:** Čudovnyj, Goliáš, LE-8711, Tilton;

**Group 11:** Forum, Jvan-Sin, Krupnoploda, Voskij, VP-LE-12/6;

**Group 12:** Mamaia;

**Group 13:** Early Blush, Gergana, H-II45/26, Chersonskij, Konzervnyj pozdnyj, Lejara LE-386, Leskora, Ljotčik, Merkurij, Náchodský zázrak, Pacov;

**Group 14:** 5-17-103, Abu Talibu, Antonio Erani, C4R8T22, Early Gold, Litoral, Melitopolskij, Poirat, Posjolok, Saldcot, SEM Badem Eric;

**Group 15:** MK 132 Visus Foee.

**Figure 6:**
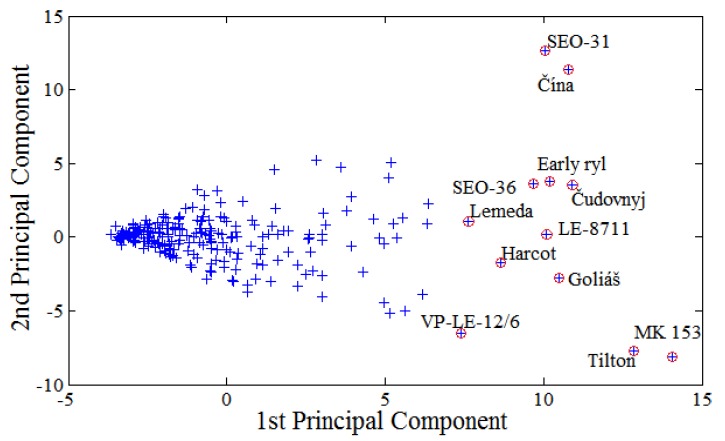
Statistical expression of extreme values of polyhenols content represented by two principal components. The marked apricot cultivars had the extreme values markedly different from the others.

Extreme divergences from cluster representation were also observed in the two-dimensional projection (Figure 6).

In this case, two principal components represent 70% of standard characters of input parameters, so, extreme values are determined more precisely. Figure 6 shows the two-dimensional expression of the content of phenolic compounds and cultivars with distant values of this parameter.

### 2.4. Determination of Antioxidant Activity

Flavonoids and phenolic acids (which belong to the polyphenols group), tocopherols, carotenoids, phosphatides, polyfunctional organic acids, ascorbic acid, which is closely connected with the glutathione-ascorbate cycle, some trace elements, such as zinc and selenium, and some enzymes participating in the enzymatic protection against reactive oxygen species are typical antioxidants found in fruits [36,72,73,74,75]. One of the methods for comparing individual fruits is determination of their antioxidant activity. It has been demonstrated that antioxidant activity depends significantly on the type of polyphenols found in fruits. There are plenty of methods for determination of antioxidant activity, based especially on the elimination of radicals (DPPH, TEAC, ORAC, PCL) or on determination of redox properties of a sample (FRAP, voltammetry, high performance liquid chromatography). It is necessary to evaluate effects of chemically different compounds based on different antioxidant mechanisms for the determination of antioxidant activity. Therefore, a combination of various methods is highly recommended [76,77]. In this study, we simultaneously used five principally different methods—DPPH, ABTS, FRAP, DMPD and Free Radical—for determination of the antioxidant activity of apricot fruits. The results obtained were recalculated to gallic acid equivalents (GAE).

**Figure 7:**
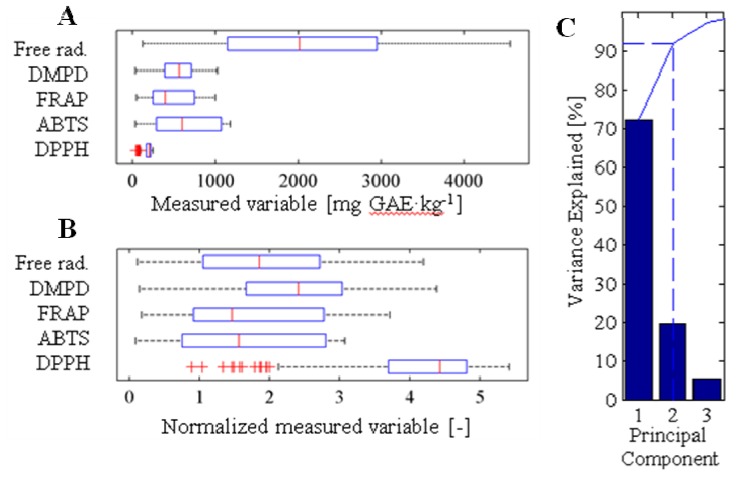
Statistical evaluation of antioxidant activities measured by Free radicals, DMPD, FRAP, ABTS and DPPH. Box plot graphs demonstrating rate of dispersions of individual methods for: (**A**) original measured data; (**B**) and for normalised values according to standard deviation; (**C**) representation of obtained principal components.

The lowest antioxidant activity values assayed by the DPPH method were determined in the Sundrop and Strepet cultivars; on the other hand, the highest values were found in the China and Colomer Nativ cultivars. The ABTS test revealed that the lowest values were determined in the LE 982 and Chvan-Shi-Cong cultivars, and the highest ones in the Doc. Blatný and Náchodská krajová cultivars. The FRAP method showed the lowest values for Leskora and Strepet cultivars and the highest for the Náchodský zázrak and LE-5832 cultivars. The DMPD method based on the scavenging of free radicals demonstrated the lowest values for the M 56 and Leskora cultivars and the highest for Colomer Nativ and LE-946. NJA-1 and Lefrosta showed the lowest values in the Free Radicals test, in comparison with the Favorit and Luizet cultivars with the highest activity. Figures 7A and 7B demonstrate the effect of individual methods before and after normalization using standardization of standard deviation of the resulting principal components. For comparison and mathematical processing, the above-mentioned data were analysed using PCA. Although minimization of principal components from five to three seems to be redundant, this minimization is very effective for PCA. All obtained data were converted into a two-dimensional projection, because two principal components represent 92% of all measurement results (Figure 7C). The PCA method detected a highly significant statistical correlation between the antioxidant activity values. This fact was subsequently verified by correlation analyses.

**Figure 8:**
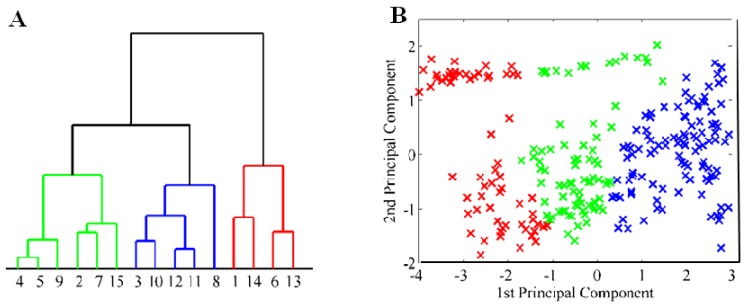
(**A**) Statistical clustering of apricot cultivars according to their antioxidant activity. All apricot cultivars were clustered into three clusters; (**B**) Three-dimensional projection of the clusters to distinguish their relations.

Effectiveness of conversion of data into analysis of two principal components was very suitable for comparison by cluster analysis (Figure 8A), demonstrating the distribution of PCA values (Figure 8B). Colour differentiation of corresponding clusters in the distribution of two primary components was almost completely geometrically separated.

All apricots were divided into 15 groups as follows. This presentation of data analysis enables determination of apricot cultivars which are similar in polyphenol compounds contents:

**Group 1:** Arzam. Aromatnyj, Harcot, H-II 19/40, In-Bez-Sin, Krymsk Medunec, LE-1402, LE-2267, LE-4725, LE-5500, Legolda, Litoral, M 56, Moi-Schva-Sin, Rival, SEO-116, Veselka 74/14, Vivagold;

**Group 2:** Antonio Erani, Bai-Gon, Forum, LE-6016, LE-7150, MK 132 Visus Foee, NJA-2, VP-LE-12/6;

**Group 3:** 5-17-103, Aviator, Colov 19, DA-YU-BADA, Exherova, Gvardějskyj, Harglow, I 51/00, Krupnoploda, LE-3709, LE-6283, LE-858, Ledana, Lenova, Ljotčik, M 47, Machová, Martina Bassi, Melitopolskij, Merkurij, Nansy Aprikose, NJA-35, NJA-55, Nugget, Poirat, Poyer, Priusaděbnyj, Reliable, Reumberto, Riland, Sem Badem Eric, SEO-111, SEO-74, Sungiant, Šalah, Štepnjak Oranževyj, Velvaglo, Vnuk Krasnoščokogo, VP-LE1212;

**Group 4:** Achrori, Early Blush, H-I-9149, Klosterneuburger, Lepana, Ligeti Orias, Markulesti, Marlen, Murfatlar, Murgab, Polonais, Roxana;

**Group 5:** Blenheim, Goldcot, Kostuřenskyj, Lakanyj, Polgocvetna, Rouge de Fourner, Scout, Vinoslivij;

**Group 6:** 6-10-45A, Abu Talibu, Ananasnyj Čjuripinskij, Bergeron A114, Goliaš, Churmai, Chvan-Shi-Cong, LE-946, LE-994, Lefrosta, Lejara LE -386, maďarská235C, Moldavský krup, Pacov, Vesprima, Voskij;

**Group 7:** Amos;

**Group 8:** Aurora, Cegledi Bilbor, Curtis, Early Ryl, Festivalna, Gulia, Hacihaliloglo, Hendersen, H-II 45/26, Krasnošč Nikitskij, LE-3204, Náchodský zázrak, Oranževo Krasnyj, PLM.78FIŠR, Pozdní chrámová, Rotmaler, Sabinovská LE-220, Saldcot, Seo, Sosed, Sunglo, Užgorod, Vardaguin Vagdaas, Vebama, Velikyj, Velita, Wenatchee, Zorkij;

**Group 9:** A-II 25/65, Alfred, Apricos von nansy, Blenheim Orange, California, Cocov, Čína, Čudovnys, Dacia, Docteyr Maccle, Fruhe von Kitsee, Goldrich, H-14/25, Hargrand, Harval, Ivone Liverani, Jvan-Sin, Keczli Mete Rosen, LE-11/91, LE-5306, LE-7463, Lebeza, Lefreda LE-831, Magiar Kajszii, Manicot, Mold Olimpik, Nikitskij, Pastyrik, Pentagon, Poljus Južnyj, Rouge de Sarnhac, Ružová Ranná, SEO-104, SEO-40, SEO-41, SEO-94, Stelar, VS22/23, VS-23/164, Zard;

**Group 10:** 5-8-8, Ackermann, Ansu, Čačansko zlato, Early Gold, Farmingdale, Helena de Rousilion, Chersonskij, Kospotěnskij, Kostinskij, Mai-Huang, Posjolok, Pretendent, TDB, Tilton, Vyndrop;

**Group 11:** Colomer Nativ, Gergana, Leronda, Tabu, Vesna;

**Group 12:** Agat, Doc. Blatný, Favorit, H-II 16/1, Krupnoplodá II, LE-5832, LE-995, Lemeda, Lerosa, Luizet, Nachodská Krajová, Orange red, SEO-31, SEO-36;

**Group 13:** Celgledi Biror, Goldtropfen, Iskra, Jubilejnyj, Konzervnyj pozdnyj, Labert in 4NO, LE-985, Leala, Mamaia, Marena, Merculešti 17/2, Perfection, SEO-105, Sungold, Veecot, Volšebnyj, VP-LE-118;

**Group 14:** Beliana, C4R8T22, Chvang sin, Julskij, LE-1075, LE-982, Leskora, Mongold, NJA-1, Rodina, Scana, Strepet, Sundrop;

**Group 15:** Efekt-22/7, Lajcot, LE-5854, LE-8711, Rouge de Rive Saltes.

**Figure 9:**
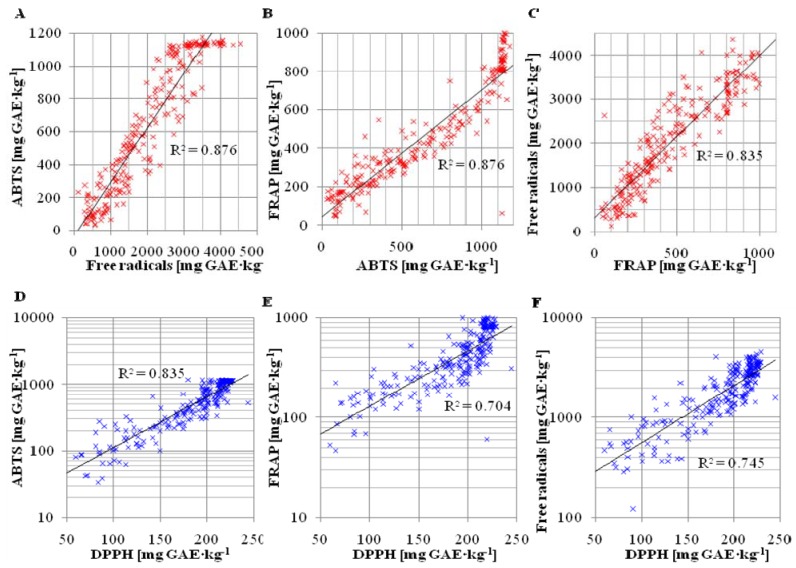
Correlograms between methods for determination of antioxidant activity as follows: (**A**) ABTS × Free radicals; (**B**) FRAP × ABTS; (**C**) Free radicals × FRAP; (**D**) ABTS × DPPH; (**E**) FRAP × DPPH and (**F**) Free radicals × DPPH.

Explanation of the obtained data based on cluster analysis may be carried out by monitoring dependences between individual methods. Because PCA is based on searching for linear dependencies between input parameters, it is effective to prepare PCA from correlograms for individual parameters. In the first step, the rate of cross-correlation of individual methods was determined. We found out that three of them (ABTS, FRAP and Free radicals) had a linear dependence with each other. Their rate of correlation was always higher than 90%. The individual correlograms are shown in Figures 9A, B and C, with linear regression and confidence coefficient 83%. In addition, the values of these three methods had an exponential dependence on values obtained using the DPPH assay. Figures 9D, 9E and 9F show correlograms with linear regression (confidence coefficient 83%) projected for logarithmic ration scale for DPPH values. Rate of correlation between these four methods is not lower that 75%. The DMPD assay was the only method used without any correlation to other used methods. Rate of correlation is about 20% and the dependence of DPMD assay on the Free Radicals method demonstrated no significant tendency. Correlations of other methods with the DMPD results showed the similar random distribution. Despite this fact, a high rate of correlation for the four used methods verifies the quality and effectiveness of PCA analysis for the antioxidant activity of apricot fruits.

The summary of values of correlation coefficient between individual methods used for determination of antioxidant activity is shown in Table 2. High antioxidant activity of apricot fruits has been confirmed by many authors assuming correlations between content of polyphenols and antioxidant activity [28,78,79]. In this study, previously published assumptions have been confirmed experimentally as well as by statistical analysis.

### 2.5. Determination of Total Polyphenols

More than 5,000 compounds of natural origin, especially secondary metabolites, with chemoprotective effects are known. These compounds include phenolic and polyphenol compounds [80,81,82]. They play important role not only in human health, but also, in the case of apricots, in resistance to pathogens, such as the viral disease *Plum pox potyvirus* [83,84]. In this study, we focused on determination of total contents of phenolic/polyphenolic compounds. In an ideal case, it would be useful to determine these compounds individually by the use of HPLC. On the other hand, there are some important disadvantages such as the fact that their separation may be very complicated, and, in addition, the structures of some polyphenols remain unknown. In this view, determination of individual compounds in fruits is almost impossible. Determination of individual phenols as antioxidants may be considered supplemental, but interesting because of their effects. Due to above-mentioned facts, we used a method for determination of total phenols content known as the Folin-Ciocalteu method based on an oxidation-reduction reaction whereby phenols are oxidized in an alkaline medium and contemporaneously a phosphotungstic-phosphomolybdene complex is reduced to a blue one [85]. Polyphenol compounds are reducing reagents, which react together with ascorbic acid, tocopherols and carotenoids with the Folin-Ciocalteu reagent. Due to this fact, results of these determinations are usually misrepresented. This misrepresentation cannot be eliminated, but must be carefully considered [86]. Values of total polyphenols in the investigated apricot cultivars ranged from 99 to 1,460 mg·kg^−1^ of gallic acid equivalent. The lowest values were determined in cultivars LE-858 (99 mg GAE·kg^−1^) and Docteyr Maccle (101 mg GAE·kg^−1^), the highest in the cultivars Favorit (1426 mg GAE·kg^−1^) and LE-7150 (1,398 mg GAE·kg^−1^). The average value was about 485 mg GAE·kg^−1^. The obtained data provide information about quantitative polyphenol content in individual samples.

**Figure 10:**
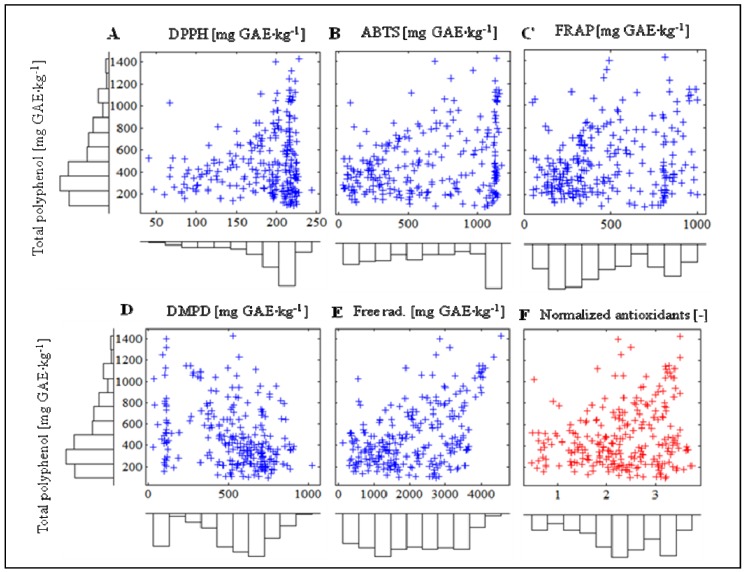
Histograms of distribution of values of antioxidant activity related to total polyphenol values.

In the next step, the relation between total polyphenol content (TP) and antioxidant activity was investigated. We found no correlation between antioxidant activity and total polyphenol content. The monitoring of distribution of values of individual methods with respect to TP distribution had higher significance. Figures 10A–F show the distribution of values for individual measurements with marginally marked histograms. Histograms have the same rate of step adjusted to the 15 levels, which quantify dispersion of values of individual methods. This fact enables evaluation of equal distribution of individual methods. In this data analysis, the DMPD method shows significant differences in comparison with other methods. In addition, the DPPH method demonstrates exponential character in its distribution of values. The last figure from this set (Figure 10F) points at a dependency of TP on the value of antioxidant activity obtained by normalization and averaging of individual values. Normalization enabled us to obtain an approximately equitable distribution of values for antioxidant activity.

## 3. Experimental

### 3.1. Biological Samples

Two hundred and thirty nine apricot cultivars (*Prunus armeniaca* L.) were used in this study. Plants were cultivated in Lednice (climatic area T4), South Moravia, Czech Republic. Fruits were harvested at consumption ripeness between the 1st July and the 15th August 2010. Fruits yields varied from 10 to 50 kg per tree. Agronomical engineering details were as follows: 15.3.2010—fertilizer *Yarakomplex* (NPK type, Yara Norge AS, Norway), dosage 120 kg·ha^−1^; 1.4.2010—fertilizer *Bortrac* (boron fertilizer, Yara, UK), dosage 200 g·ha^−1^; 30.5.2010—urea fertilizer (MJM Litovel a.s., Czech Republic), dosage 300 kg·ha^−1^; 5.6.2010—fertilizer *Zintrac* (Yara, UK), dosage 1.5 kg·ha^−1^, 10.6, 10.7, 10.8 fertilizer *Sampi* (Sampi, Indonesia), dosage 15 kg·ha^−1^. Decade rainfall totals and average temperature from 1.5.2010 to 20.8.2010 are shown in Table 3.

### 3.2. Sample Preparation

Fruits samples (approx. 2 g) were quantitatively transferred into a grinding mortar and homogenized with water (8 mL, ACS purity, Sigma Aldrich, USA). Homogenised samples were quantitatively transferred into test tubes and under the same laboratory conditions were vortexed for 30 min. (VORTEX Genius 3, IKA, Germany). Samples were subsequently centrifuged for 30 min at 16,400 rpm (Eppendorf 5430R, Czech Republic). After centrifugation, supernatants were obtained and used for individual analyses. Despite the fact that organic solvents like methanol or ethanol belong to the group of most used solvents for polyphenols extraction, water was chosen because of necessity of complex analysis [87].

For amino acids determination, dried and pulverized sample (0.025 g) was mixed with HCl (6 M, 0.5 mL). Prepared sample was transferred into a MG5vial and placed in a 64MG5 rotor. The rotor with samples was placed in microwave system and subsequently mineralized in a Multiwave3000 microwave apparatus (Anton-Paar GmbH, Germany). Conditions of microwave decomposition were: power 100 W, ramp 10 min, hold time 140 min, cooling time 10 min.

### 3.3. pH Determination

The pH value was measured using an inoLab Level 3 with terminal Level 3 (Wissenschaftlich-Technische Werkstätten—WTW, Weilheim, Germany), controlled by a personal computer program (MultiLab Pilot; WTW). The pH-electrode (SenTix-H, pH 0–14/3M KCl) was calibrated with a set of buffers (WTW). Deionised water underwent demineralization by reverse osmosis using an Aqua Osmotic 02 instrument (Aqua Osmotic, Tisnov, Czech Republic) and then it was subsequently purified using Millipore RG (Millipore Corp., USA, 18 MΏ)—MiliQ water.

### 3.4. Analysis of Amino Acids by Ion Exchange Chromatography

An AAA 400 (Ingos, Czech Republic) liquid chromatography apparatus was used for determination of amino acids. The system consists of a glassy filling chromatographic column and steel precolumn, two chromatographic pumps for transport of elution buffers and derivatization reagent, cooled carousel for 25 test tubes of 1.5–2.0 mL volume, dosing valve, heating reactor, VIS detector and cooled chamber for derivatization reagent. The glassy chromatographic column (i.d. 3.7 mm and 350 mm length was filled with LG ANB strong catex in sodium cycle (Spolchemie, Czech Republic) with particles of average size of 12 µm and netting of 8%. The glassy column was tempered by a thermostat working in the 35–95 °C range. The precolumn was filled by LG KS0804 ionex (Ingos, Czech Republic). Chromatographic columns for transfer of elution buffers and derivatization reagent are able to work at flows of 0.01–10 mL·min^−1^ under a maximum pressure of 40 MPa. Volume of injected sample was 100 µL, with an accuracy of application RSD of about 1%. A two-channel VIS detector with a 5 µL flow volume cuvette was operated at wavelengths of 440 and 570 nm. Ninhydrin solution (Ingos, Czech Republic) was used as derivatization reagent. Ninhydrin was dissolved in solution containing 75% (*v*/*v*) of the organic solvent methyl cellosolve (Ingos, Czech Republic) and 25% (*v*/*v*) of 4 M acetate buffer (pH 5.5). SnCl_2_ (Lachema, Czech Republic) was used as reducing agent. Derivatization reagent was stored for the whole time under an inert atmosphere (N_2_) with cooling at 4 °C. Amino acids elution was carried out according to Table 4 and Table 5. During the analysis, the flow of mobile phase was set at 0.3 mL·min^−1^ under a pressure ranging from 4.5 to 6.0 MPa. Reactor temperature was set to 120 °C.

### 3.5. HPLC Profile of Selected Polyphenols

For the determination of the HPLC profiles of individual apricot cultivars, high performance liquid chromatography with electrochemical and UV-VIS detection was used. The system consisted of two Model 582 ESA chromatographic pumps (ESA Inc., Chelmsford, MA, USA) with a working range from 0.001 to 9.999 mL·min^−1^ and a Zorbax SB C18 (150 × 4.6; size of particles 5 µm, Agilent Technologies, USA) reverse phase chromatographic column. For UV detection, a Model 528 ESA UV detector was used. A twelve-channel CoulArray detector (ESA) was used for electrochemical detection. Samples were injected automatically by an autosampler (Model 542, ESA), which has incorporated a thermostated space for a column. The chromatographic conditions can be found in Zitka *et al.* [88].

### 3.6. Determination of Antioxidant Activity

An automated BS-400 spectrophotometer (Mindray, China) was used for determination of antioxidant activity according to the following protocols.

#### 3.6.1. Determination of antioxidant activity by the DPPH test

A volume of DPPH^•^ reagent (200 μL) [89] was incubated with sample (20 μL). Absorbance was measured after 15 min of incubation at λ = 510 nm. For calculating the antioxidant activity value of absorbance of reagent itself (A_0_) and the value of absorbance after 15 min of incubation (A_15_) were used. Resulting value was calculated according to the formula: A = A15 − A0.

#### 3.6.2. Determination of antioxidant activity by the ABTS test

A volume of ABTS^•^ reagent (245 μL) [89] was pipetted into a plastic cuvette with subsequent addition of sample (5 μL). Absorbance was measured at λ = 670 nm after 15 min. For calculating the antioxidant activity, we used the value of absorbance of reagent itself (A_0_) and the value of absorbance after 15 min of incubation (A_15_). Resulting value was calculated according to the following formula: A = A15 − A0.

#### 3.6.3. Determination of antioxidant activity by the FRAP method

A volume of FRAP^•^ reagent (245 μL) [89] was pipetted into a plastic cuvette with subsequent addition of sample (5 μL). Absorbance was measured at λ = 578 nm after 15 min. For calculating the antioxidant activity, we used the value of absorbance of reagent itself (A_0_) and the value of absorbance after 15 min of incubation (A_15_). Resulting value was calculated according to the following formula: A = A15 − A0.

#### 3.6.4. Determination of antioxidant activity by the DMPD method

A volume of DMPD^•^ reagent (200 μL) [89] was pipetted into a plastic cuvette. Then, sample (5 μL) was added. Absorbance was measured at λ = 510 nm for 15 min. For calculating the antioxidant activity, the value of absorbance of reagent itself (A_0_) and the value of absorbance after 15 min of incubation (A_15_) were used. Resulting value was calculated according to the following formula: A = A15 − A0.

#### 3.6.5. Determination of antioxidant activity by the free radicals method

Into a plastic cuvette, a volume of prepared reagent (200 μL) [89] was pipetted. Further, sample (8 μL) was added. Absorbance was measured at λ = 450 nm for 15 min. For calculating the antioxidant activity, we used the value of absorbance of reagent itself (A_0_) and the value of absorbance after 15 min of incubation (A_15_). Resulting value was calculated according to the following formula: A = A15 − A0.

#### 3.6.6. Determination of total content of polyphenols

The Folin-Ciocalteu method, based on the reduction of a phosphotungsten-phosphomolybdate complex by phenols to blue reaction products, was used for determination of phenolic compounds. Sample (0.5 mL) was pipetted into cuvette and diluted with ACS water (1.5 mL). Subsequently, Folin-Ciocalteu reagent (50 μL) was added and the solution was incubated at 22 °C for 30 min. The absorbance was measured using a dual-beam SPECORD 210 spectrophotometer (Carl Zeiss Jena, Germany) at a wavelength λ = 670 nm against blank (all chemicals without a sample or gallic acid) according to [86]. The absorbance was measured in triplicate. Results were expressed as equivalents of gallic acid in mg·100 g^−1^. The method was calibrated on the well-known phenolic compound gallic acid.

### 3.7. Mathematical and Statistical Analysis

Mathematical and statistical analysis of experimental data was carried out with MATLAB®, Version 7.9.0.529 (R2009b, MathWorks Inc., Natick, MA, USA). Multidimensional analysis including both principal components analysis and cluster analysis were used for statistical evaluation of data due to large set of the results obtained. *PCA calculation procedure*. In first step, we perform principal components analysis (PCA) on the n-by-p input data matrix X, and returns the principal component coefficients, also known as loadings. Rows of X correspond to apricots, columns to variables. Result is a p-by-p matrix, each column containing coefficients for one principal component. The columns are in order of decreasing component variance. The second output, scores, contains the coordinates of the original data in the new coordinate system defined by the principal components. This output is the same size as the input data matrix. The scores are the data formed by transforming the original data into the space of the principal components. The values of the vector latent are the variance of the columns of SCORE. The third output, variances, is a vector containing the variance explained by the corresponding principal component. Each column of scores has a sample variance equal to the corresponding element of variances. The last output of the PCA function is Hoteling’s parameter T^2^, a statistical measure of the multivariate distance of each observation from the centre of the data set. This is an analytical way to find the most extreme points in the data. Finally, we use the biplot function to help visualize two or three the principal component coefficients for each variable and the principal component scores for each apricot in a single plot.

*CA calculation procedure*. We use the *pdist* function to calculate the distance between every pair of objects (apricots) in a data set. We compute the Euclidean distance between pairs of objects in m-by-n data matrix X. Rows of X correspond to observations, and columns correspond to variables. D is a row vector of length m(m–1)/2, corresponding to pairs of observations in X. D is dissimilarity matrix for next clustering. The *pdist* function calculates the Euclidean distance between objects, but we can specify one of several other options. We use the standardized Euclidean, so each coordinate difference between rows in X is scaled by dividing by the corresponding element of the standard deviation. The next step is agglomerative hierarchical clustering to tree by UPGMA uses the smallest distance between objects in the two clusters.

## 4. Conclusions

In our experiments, we focused on the comprehensive study of the less common parameters of 239 apricot fruit cultivars, which represent the Czech Republic Gene Pool of this fruit species. Bioinformatics evaluation of data using cluster analysis and principal component analysis demonstrates the possibility to process such a large data set and to find some correlations. This evaluation enabled proposition of clusters, where individual apricot cultivars were classified according to their similarity in the content of target molecules. Used methods seem to be very convenient for evaluation of biological and nutrition value of the apricot cultivars.

We clustered all cultivars into 3 × 15 groups according to their amino acids content, polyphenols content and antioxidant activity. Such comprehensive characteristic of gene pool has not been done before and could be used as a basis for further nutritional evaluation of these cultivars. Moreover, the bioinformatics treatment of our data revealed some connections between all three determined parameters. LE cultivars have been clustered into the similar groups, which clearly show on the connection between antioxidant activity, content of polyphenols and amino acids. On the hand, there have been other cultivars without any relation of these three parameters. Based on our results, one may choose some cultivars and characterized them from the point of view of other parameters such as resistance against some viruses.

## Figures and Tables

**Table 1:** Average concentrations of amino acids per one apricot cultivar (expressed as an average of all 239 apricot cultivars). Values are expressed in mmol·L^−1^.

Amino acid	Asp	Thr	Ser	Glu	Pro	Gly	Ala	Val	Ile	Leu	Tyr	Phe	His
**Average Concentration [mM]**	593	4,526	557	288	416	3,877	3,385	396	280	156	162	2,115	184

n = 3; RSD = 2%.

**Table 2:** Values of correlation coefficients for determination.

	ABTS	FRAP	Free radicals	DMPD
**DPPH**	0.821	0.750	0.780	0.290
**ABTS**	**—**	0.936	0.936	0.108
**FRAP**	**—**	**—**	0.914	0.110
**Free radicals**	**—**	**—**	**—**	0.221

**Table 3:** Decade rainfall totals and average temperature from 1.5.2010 to 20.8.2010.

Decade	Decade rainfall totals (mm)	Average decade temperature (°C)
10.5.2010	44	15
20.5.2010	50	12
30.5.2010	39	16
10.5.2010	40	19
20.6.2010	41	19
30.6.2010	1	20
10.6.2010	5	22
20.7.2010	58	25
30.7.2010	45	20
10.8.2010	39	20
20.8.2010	23	21

**Table 4:** Buffers for chromatographic determination of amino acids.

	Buffer Number			
**Compound (g per 1 L)**	1	2	3	4
Citric acid	11.1	10.0	7.5	–
Trisodium citrate	4.0	5.6	9.1	19.6
Sodium chloride	9.3	8.4	18.0	52.6
Boric acid	–	–	–	2.1
Thidiglycol	2.5	2.5	2.5	–
Sodium hydroxide	–	–	–	0.5–3.0
pH	2.7	3.0	4.3	7.9

**Table 5:** Chromatographic gradient.

Time (min)	Column temperature (°C)	Buffer number
0	62	1
7	62	2
20	62	2
31	60	3
43	50	4
50	74	4
57	74	4
65	74	4
66	80	6
73	80	1
76	80	1
79	80	1
83	62	1
84	62	1
86	62	1
90	62	1

## Supplementary Materials

Supplementary materials can be accessed at http://www.mdpi.com/1420-3049/16/9/7428/s1.
